# Thank You to Our Reviewers

**DOI:** 10.1097/MD.0000000000009573

**Published:** 2018-01-05

The *Medicine*^*®*^ Editorial Office would like to thank all of our expert volunteers for their continued devotion and generosity to the journal. Peer review is an essential part of publishing high quality content and their contributions and feedback are fundamental to the process.

As a sign of our appreciation we want to recognize all of the reviewers who contributed to the journal in 2017. To all of our reviewers, we are grateful for your contributions and look forward to working with all of you again in 2018!

Best Regards,

Tom Pacific

Publisher

Bektas A

Bonifaz A

Jawhar A

Matsuada A

Oishi A

Ozyilmaz A

Zuchetto A

Pazarli A Cemal

Lee A Leum

Kersten A.

Buckland Aaron

Moberly Aaron

Ward Aaron

Contractor Aashish

Elsherif Abdelhalim

S. Gounni Abdelilah

Bellou Abdelouahab

Oussalah Abderrahim

Qureshi Abdul

Sulaiman Abdul Razak

Banday Abdul Rouf

Cingü Abdullah

Tuncez Abdullah

Abhishek Abhishek

Kumar Abhishek

Najmi Abul K.

Susmarski Adam

Javed Adil

Mardinoglu Adil

Surendiran Adithan

Sinha Aditi

Karaibrahimoğlu Adnan

Mir Adnan

Sfera Adonis

Elliott Adrian

Wagg Adrian

Tzioufas Ag

Muñoz Sanz Agustin

Najjar Ahed

Abdel-Razik Ahmed

Elshamy Ahmed

Pasha Ahmed

Siam Ahmed

Besir Ahmet

Eroglu Ahmet

Ikiz Ahmet

Issin Ahmet

Uçar Ahmet Deniz

Eskazan Ahmet Emre

Qiao Aijun

Fang Aiqin

Hailder Ajay

Yadlapati Ajay

Vikram Ajit

Siriwardena Ajith

Ajmera Akash

Ergonen Akca Toprak

Chaurasia Akhilanand

Turna Akif

Sabarudin Akmal

Boussuges Alain

Meyer Alain

Baer Alan

Cook Alan

Fotoohi Alan

Needle Alan

Zubair Alauldeen

Todorova Albena

Varga Albert

Ramelet Albert Adrien

Avolio Alberto

Farias Alberto

Fernandez-Medarde Alberto

Polimeni Alberto

Tedeschi Alberto

Jorge-Mora Alberto Agustín

Marrone Aldo

Chade Alejandro

Horga Alejandro

López-Magallon Alejandro

Moreno Alejandro

Truszczyńska Aleksandra

Codegone Alessandra

Antonelli Alessandro

Cavicchioli Alessandro

Soria Alessandro

Alunno Alessia

Paffoni Alessio

Isaacs Alex

Nunes Alex

Sweeney Alex

Ng Alex Lk

Kokkinos Alexander

Novotny Alexander

Rodriguez Alexander

Rösch Alexander

Winter Alexander

Wolanskyj Alexandra

Webb Alexandra (Sasha)

Laedermann Alexandre

Daponte Alexandros

Samartzis Alexandros

Otrakji Alexi

Ulrich Alexis

Reginelli Alfonso

Canbay Ali

Cetinkaya Ali

Zaid Ali

Safarpour Ali Reza

Pizent Alica

Sales-Galan Alicia

Gunes Alime

Kahraman Alisan

Sanders Alison

Van Dyke Alison

Khurana Alkesh

Sahin Alparslan

Sahin Alpaslan

Tanoglu Alpaslan

Abarzua Alvaro

Simanek Amanda

Gogovor Amede

Puranik Ameya

Hamidieh Amir Ali

Mozafarykhamseh Amirhossein

Alwaal Amjad

Kothekar Amol

Coira Amparo

Barczak Amy

Kennedy Amy

Zeng An

Carvalhosa Ana

Charrua Ana

Gutiérrez Ana

Póvoa Ana

Friginal-Ruiz Ana Belén

Nastri Ana Catharina

Orbai Ana-Maria

Domijan Ana-Marija

Štafa Anamarija

Bajpai Anamika

Antoniadou Anastasia

Dede Anastasia

Lymperopoulos Anastasios

Streinu-Cercel Anca

Ignat Ancuta

Boyd Anders

Kaelin Andre

Angelini Andrea

Calcagno Andrea

Grillo Andrea

Lembo Andrea

Picchianti-Diamanti Andrea

Ruzzenente Andrea

Witzel Andrea

Müller Andreas

Wannhoff Andreas

Koptioug Andrei

Fernandez-Codina Andreu

Daly Andrew

Griffith Andrew

Kan Andrew

Mammen Andrew

Murphy Andrew

Petroll Andrew

Naska Androniki

Schmidt André

Henao-Martínez Andrés

Ruf Andrés Eduardo

Grumach Anete

Sun Ang

Guerrero Angel L.

Johnson Angela

Quirino Angela

Debs Angelica.

Janitzky Angelika

Di Iorio Angelo

Pompucci Angelo

Gaffo Angelo L

Asúnsolo Ángel

Sari Anggraini

Seetharam Anil

Gupta Anil Kumar

Dey Anindya

Jellad Anis

Thachangattuthodi Anish

Parihar Anit

Gera Anjali

Danoff Ann

Raes Ann

Cantarutti Anna

Lo Bue Anna

Nowinska Anna

Ralph Anna

Serafini Anna

Zygogianni Anna

Calavalle Anna Rita

Amstrup Anne

Ducloy-Bouthors Anne-Sophie

Roos Annerine

Cai Anping

Kuang Anren

Teoh Anthony

Finianos Antoine

Castagna Antonella

Niedzielski Antoni

Bianco Antonino

Tuttolomondo Antonino

Andreacchio Antonio

Giuliani Antonio

Palazón-Bru Antonio

Paoli Antonio

Ponzetto Antonio

Russo Antonio

Secchi Antonio

Michalopoulos Antonios

Vlahos Antonios

Papadopoulos Antonis

Silva Antônio Eduardo

Singh Anup

Shirali Anushree

Damle Archana

Agrawal Archi

Pandey Arjun

Ng Arlene

Ahmed Armin

Rashidi Armin

Kamman Arnoud

Templeton Arnoud J

Cicero Arrigo

Tang Arthur

Slomka Artur

Chogle Arun

Ramanujam Arvind

Varghese Asha

Gandhe Ashish

Khandelwal Ashish

Suri Ashish

Vincent Ashley

Sood Ashwani

Kalshetty Ashwini

Thomas Astrid

Demir Atakan

Tragiannidis Athanasios

Jindal Atul

Padole Atul

Guffroy Aurélien

May Austin J

Kambadakone Avinash

Andres Axel

Torun Ayşe Nur

Alsebaey Ayman

Kefeli Ayse

Salihoglu Ayse

Khalili Azadeh

Tabari Azadeh

Rodríguez-Alfonso B

Von Scholten B J

Abdinia Babak

Bijin Babitha

Gupta Bal Kishan

Parameswaran Balaji

Singh Baljinder

Firouzé Bani-Sadr

Sis Banu

Cui Baojuan

Zhong Baoliang

Li Baosheng

Li Baosheng

Sevinç Barış

Parolini Barbara

Erdal Barbaros

Ikitimur Baris

Malpani Basant

Pintaudi Basilio

Haerian Batoul

Müller-Stich Beat

Tiftikcioglu Bedile Irem

Hassani-Mahmooei Behrooz

Wang Bei

Xu Beibei

Sun Beicheng

Tanriover Bekir

Prieto Belen

Zarzaur Ben

Maliakkal Benedict

Almirante Benito

De Celis Benito

Buecking Benjamin

Fairfax Benjamin

James Benjamin

Longo-Mbenza Benjamin

Misselwitz Benjamin

Dervaux Benoit

Voisin Benoit

Haileselassie Bereketeab

Haileselassie Bereketeab

Cohen Bernard

Schaller Bernhard

Lopansri Bert

Taylor Beth

Rao Bhaskar

Gajera Bhavin

Naik Bhiken

Patel Bhupeshwari

Cheng Bi-Hua

Bianba Bianba

Bianco Bianca

Xu Biao

Goran Bicanic

Oz Bilgehan

Ince Bilsev

Fang Bin

Li Bin

Lu Bin

Wang Bin

Yan Bin

Yi Bin

Yu Bin

Cheng Binbin

Han Bing

Zhou Bing

Shi Bingyin

Nepal Binod

Savani Bipin

Luo Biquan

Ozcakar Birsin

Fu Bishi

Wen Bixiu

Weiß Björn

Løfgren Bo

Ma Bo

Yuan Bo

Wang Boliang

Firestein Bonnie

Teo Boon Wee

Bernhardt Boris C.

Hale Braden

Mcgwire Bradford

Singh Brajesh

Rabeler Brandon

Paty Breay

O’Connell Brendan

Gibbons Brent

Boulay Brian

Egleston Brian

Gulson Brian

Morris Brian

Silver Brian

Galling Britta

Shaw Bronwen

Crookes Bruce

Baroni Bruno

Megarbane Bruno

Nascimento Bruno

Saragiotto Bruno

Sedassari Bruno

Spire Bruno

Trimarco Bruno

Satar Bulent

Yavuz Burcu Balam

Jang Byoung Kuk

Shin Byung-Cheul

Park Byung-Joo

Kim Byung-Su

Koehl Bérengère

Altunoluk Bülent

Zhang C

Yu C-L

Bock C-Thomas

Chan C.-C.

Cheung C.W.

Millar Cameron

Gallo Camila

Laurent Camile

Tincati Camilla

Frances Camille

Perchoux Camille

Yaldiz Can

Yerebakan Can

Ceran Candemir

Domenech Carine

Chen Carl

Kendall Carl

Lavie Carl

Pepine Carl

Buonerba Carlo

Lomonte Carlo

Perricone Carlo

Trani Carlo

Cervera Carlos

De Vasconcelos Carlos

Guillen Astete Carlos

Gómez-Escalonilla Carlos

Lozoya-Ibáñez Carlos

Paiva Carlos

Ordás Carlos M.

Lunghi Carlotta

Evans Carlton

Pezaro Carmel

Serna-Candel Carmen

Tomasetti Carmine

Moreira Carolina

Palmela Carolina

Taylor Caroline

Berryman Carolyn

Santos Catarina

Mammina Caterina

Buchan Catharine

Cornu Catherine

Dube Catherine

Ong Catherine

Staton Catherine

Choong Cathy

Rozmus Cathy

Zheng Ce

Costiniuk Cecilia

Sun Celi

Dane Cem

Ertan Cem

Ucgul Atilgan Cemile

Pant Chaitanya

Venkateswarlu Chamcha

Venkateswarlu Chamcha

Bal Chandrashekhar

Gong Chang

Kim Chang Woo

Jenq Chang-Chyi

Kuo Chang-Fu

Lu Chang-Hsien

Lee Changhyun

Zhang Changqing

Wang Changsong

Wei Changyong

Park Chanhyun

Fan Chao

Fang Chao

Huang Chao

Liu Chao

Hsu Chao Wei

Hung Chao-Hung

Mandros Charalampos

Brennan Charles

Everett Charles

Adamczick Charlotte

Markert Charlotte

Sheu Chau-Chyun

Hu Chaur-Jong

Lin Che-Hsuan

Ahmed Chebil

Foo Chee Yoong

Sng Chelvin

Chen Chen

Liang Chen

Mao Chen

Zhang Chen

Zhang Chen

Chung Chen-Chih

Cherng Chen-Hwan

Feng Chenchen

Huang Chenfei

Huang Cheng

Liu Cheng

Yang Cheng-De

Lee Cheng-Hung

Lin Cheng-Jui

Lin Cheng-Wen

Peng Cheng-Yuan

Hu Chengbin

Meng Chengbo

Zhu Chengcheng

Chen Chengpeng

Gao Chenxi

Tian Chenxi

Dallal Cher

Frydman Cheryl

Hung Chi-Chih

Tseng Chi-Chuan

Chiang Chi-Ling

Huang Chi-Ling

Dai Chia Yen

Chang Chia-Chu

Li Chia-Hsiang

Lin Chia-Hung

Chung Chia-Min

Chao Chia-Ter

Lu Chia-Wen

Hsu Chia-Yang

Chu Chia-Yu

Lazzeri Chiara

Nduka Chidozie

Taniguchi Chie

Khor Chiea Chuen

Lu Chieh-Sheng

Lee Chien-Chang

Huang Chien-Cheng

Li Chien-Feng

Chen Chien-Hung

Chen Chien-Hung

Liao Chien-Hung

Chao Chien-Ming

Liao Chien-Wei

Su Chien-Wei

Tseng Chih -Wei

Lai Chih Hung

Kao Chih-Chin

Chen Chih-Chung

Yeh Chih-Fan

Chuang Chih-Hsien

Hsu Chih-Hsin

Lee Chih-Hsin

Chi Chih-Yu

Huang Chihyuan

Terao Chikashi

Huang Chin-Chou

Su Chin-Hui

Chong Ching Ning

Cheng Ching-Feng

Liu Ching-Feng

Tseng Ching-Min

Hsu Ching-Sheng

Mani Chinnadurai

Ma Chiyuan

Wu Cho-Kai

Chen Chong

Gardiner Chris

Panopoulos Chris

Verhofstede Chris

Johansson Christer

Cordano Christian

Garcia Christian

Oberkofler Christian

Rupp Christian

Toso Christian

Bamia Christina

Mehner Christine

Liebetrau Christoff

Schliemann Christoph

Schmaderer Christoph

Auvenshine Christopher

Hocking Christopher

Nettles Christopher

Roman Christopher

Rose Christopher

So Christopher

Mavrommatis Christos

Rountas Christos

Triantos Christos

Papadopoulou Chryssanthi

Yen Chuan-Min

Wang Chuanxi

Liu Chuanxu

Yen Chueh-Chuan

Zhu Chuhong

Gao Chun

Yang Chun

Huang Chun-Che

Liu Chun-Hua

Lin Chun-Ju

Wang Chun-Meng

Liao Chun-Ta

Liu Chun-Yu

Hwang Chung-Feng

Ho Chung-Han

Li Chung-Pin

Liu Chung-Pin

Chen Chung-Yu

Bian Chunjing

Zhao Chuntao

Gao Chunxu

Lin Chuwen

Chen Chyi-Liang

Zhou Cindy

Indolfi Ciro

Gareri Clarice

Cardoso Claudia

Pisanu Claudia

Sardu Claudia

Zerbini Claudia

Iaconetti Claudio

Letizia Claudio

Mesquita Claudio

Tana Claudio

Madeddu Clelia

Mavragani Clio

Kelly Clive

Ferri Clodoveo

Niederau Cluas

Lima Cláudia

Meirelles Cláudia

Passos Clévia

Mcdowell Colleen

Keane Colm

Meng Cong

Ye Cong

Lapa Constantin

Axiotis Constantine

Langner Cord

Sunderkoetter Cord

Richardson Corey

Pedrazzani Corrado

Hileman Corrilynn O.

Mclachlan Craig

Williams Craig

Vera Cristian

Calvo Cristina

Fernández-Pérez Cristina

Reschke Cristina

Sipeky Csilla

Yegen Cumhur

Wei Cun-Sheng

Ng Curtise K.C.

Crowson Cynthia

Lemogne Cédric

Lenormand Cédric

Fernández-De-Las-Peñas César

He D

Lipsker D

Meyre D

Adamson D.Cory

Berntsen DA

Cai Dachuan

Kim Dae Jung

Thomas Dafydd G.

Aune Dagfinn

Lingzhen Dai

Tokuhara Daisuke

Yang Dajun

Ding Dale

Li Dali

Attallah Dalia

Li Dan

Wang Dan

Zhou Dang-Xia

Schumann Dania

Bunout Daniel

Cuestas Daniel

Culver Daniel

Dirkmann Daniel

Fouladi Daniel

Franzen Daniel

Markel Daniel

Olive Daniel

Reim Daniel

Ribeiro Daniel

Rushing Daniel

Mazo Daniel Ferraz De Campos

Farkas Daniel T

Balaban Daniel Vasile

Marrelli Daniele

Tang Danming

Ji Dar-Der

Albert Dara

Englot Dario

Marangoni Dario

Patel Darshan

Albuquerque David

Bearden David

Cruz David

Garcia David

García-Azorín David

Hallman David

Hart David

Mitchell David

Shitrit David

Francomano Davide

Basu Debasish

Murrell Dedee

Doshi Deepak

Sharan Deepak

Mitra Deepanjan

Das Deepika Sharma

Liang Deguang

Elgui De Oliveira Deilson

Jiang Dekejiang

Goletti Delia

Chen Demeng

Changwen Deng

Min Deng

Wu Deng-Chyang

Gao Dengfeng

Pinal Denise

Kim Deok Won

Zhang Deqiang

Chan Derek

Juneja Deven

Mccaslin Devin

Rangaswamy Dharshan

Kapoor Dheeraj

Rawat Dhwaj Bahadur

Shao Di

Boraschi Diana

Magliano Dianna

Zhong Diansheng

Colpan Oksuz Didem

Gazzolo Diego

Milani Diego

Quattrone Diego

Ripamonti Diego

Haemmerich Dieter

Schmidt Dieter

Kumar Dilip

Popkov Dimitri

Koutoukidis Dimitrios

Ntourakis Dimitrios

Paraskevis Dimitrios

Ye Dingwei

Kozyrakis Diomidis

Dellaportas Dionysios

Watson Dionysios

Maskey Dipak

Cornec Divi

Tamburrino Domenico

Tricarico Domenico

Paquin Proulx Dominic

Steubl Dominik

Dumford Donald

Redelmeier Donald

Zhou Dong

Sinn Dong Hyun

Joo Dong Jin

Kim Dong Ki

Lee Dong Ki

Lee Dong Soo

Shin Dong Yeong

Kim Dong-Jun

Chen Dong-Yi

Qi Dongfei

Kim Donghee

Zhou Dongsheng

Chen Dongshi

Fan Dongwei

Zhao Dongxin

Zhang Dongze

Sierra Dr Cristina

Armario Dr Pedro

Hilmi Dr. Ibtesam

Shenberger-Trujillo Dr. Jessica

Forland Dr. Steven

Marchaim Dror

Papanti Duccio

Tripathi Durga

Kumar Dushyant

Grooms Dustin

Demirkaya E

Murray Earnest

Jarroll Ed

Tanrikulu Simsek Eda

Overton Edgar Turner

Desapriya Ediriweera

Arany Edith

Savarino Edoardo

Abdala Edson

Suarez-Martinez Edu

Coelho Eduardo

Moffa Eduardo

Ehler Edvard

Fysh Edward

Vlachaki Efi

Flores Efren

Kapsalaki Eftychia

Grossman Ehud

Chung EJ

Dobryakova Ekaterina

Georgousopoulou Ekavi

Abduh El Banna

Carvalho Elaine

Ehrlich Elana

Kane Eleanor

Bartoloni Bocci Elena

Grebenciucova Elena

Grebenciucova Elena

Varoni Elena Maria

Kharazmi Elham

Miloslavsky Eli

Manjarrez Elias

Pace Elisabetta

Becoña Elisardo

Kontis Elissaios

Azevedo Eliza

Harris Elizabeth

Neale Elizabeth

Bartlett Ellis

Baronia-Locson Elsie

Chan Elsie

Padua Elvira

Durante-Mangoni Emanuele

Diego Emilia

Sbidian Emilie

Christenberry Emily

Erbelding Emily

Hyle Emily

Hatzipantelis Emmanouil

Kalampokas Emmanouil

Effa Emmanuel

Li Emmy

Ampil Encarnita

Dulundu Ender

Feher Eniko

Pedrol Enric

Asensio Lafuente Enrique

SotoPedre Enrique

Vingolo Enzo Maria

Yildiz Eren

Tatar Erhan

Jeziorski Eric

Jorgenson Eric

Kipnis Eric

Kirschner Eric

Senneville Eric

Lai Eric Ch

Soule Eric K

Thoomes, Erik J.

Boesen Erika

Jones Erika

Alpsoy Erkan

Turan Ersin

Schaefer Esperance

Schaefer Esperance

Gunduz Esra

Ataoğlu Esra Erkoç

Martinez Esteban

Dr. Saguil Esther

Anderson Ethan

Acar Ethem

Banu Eugeniu

Chung Eun Kyoung

Kim Eun Soo

Ageberg Eva

Feigerlova Eva

Zapata Eva

Knudsen Eva Cecilie

Pedersen Eva,

Glazer Evan

Dounousi Evangelia

Sagnelli Evangelista

Potolidis Evangelos

Voulgaris Evangelos

Janczewska Ewa

Cappello Ezio

Post F

Yalniz F. Firat

Machado Fabiana

Mesquita Barros Fabiana

Barbieri Fabio

Mangiacapra Fabio

Minutoli Fabio

Nascimbeni Fabio

Natale Fabio

Presotto Fabio

Menegaux Fabrice

Dal Farra Fabrizio

Favales Fabrizio

Edrees Fahad

Yao Fan

Tao Fang

Wang Fang

He Fanggang

Wu Fangrui

Lanternier Fanny

Kosari Farhad

Nakhoul Farid

Turkcu Fatih

Alshbool Fatima

Derbali Fatma

Petrelli Fausto

Vigna-Taglianti Federica

Salomone Federico

Chen Fei

Xiao Fei

Xie Fei

Zhao Fei

Daltoé Felipe

Hüttner Felix

Steiger Felix

Philip Femi

Liu Fen

Zhou Fen

Tseng Fen-Yu

Liu Feng

Shen Feng

Wang Feng

Yang Feng

Zhang Feng

Wu Fengchun

Jakab Ferenc

Davatchi Fereydoun

Monsell Fergal

Bellodi Schmidt Fernanda

Cabral Fernanda

Pigatti Fernanda

Martínez-Valle Fernando

Masanes Ferran

Besli Feyzullah

Serraino Filiberto

Cardoso Filipe

Valbusa Filippo

Lampe Fiona

Al-Taweel Firas

Cheng FJ

Le Ven Florent

Andrei Fodor

Sjoberg Folke

Tsuang Fon-Yih

Kavvoura Fotini

Barbic Franca

Chiodi Francesca

Sanguedolce Francesca

Viazzi Francesca

Zimetti Francesca

Bartoli Francesco

De Rosa Francesco

Dieli Francesco

Fanfulla Francesco

Panza Francesco

Passafaro Francesco

Perticone Francesco

Pichi Francesco

Ursini Francesco

Virdis Francesco

Capsoni Franco

Herbstreit Frank

Lenze Frank

Lin Frank

Liu Frank

Onishi Franz

Roedel Franz

Angoulvant François

Ris Frédéric

Deyuan Fu

Wang Fu

Chung Fu-Tsai

Qin Fujun

Atamaz Funda

Sung Fung-Chang

Certel Furkan

Wang Fuzhou

Jiménez-Jiménez Félix Javier

Palumbo G

Tilve G H

Puri G.D.

Culbert Gabriel

Valero Gabriel Romualdo

Costa Gabriela

Valentini Gabriele

Mocciaro Gabriele.

Bertino Gaetano

Santulli Gaetano

Santulli Gaetano

Kaur M.D Gagandeep

Kürklü Galip Bilen

Chen Gang

Chen Gang

Fang Gang

Li Gang

Qin Gang

Wang Gang

Zhang Gang

Huang Gangxiong

Tabacchi Garden

Brennan Gary

Prakash Gaurav

Xu Gelin

Zheng Gen

Oyama Genko

Györi Georg

Chouliaras George

Dimitriadis George

Koukourakis George

Sack George

Sakoulas George

Vassilopoulos George

Khalil George M

Gakis Georgios

Rentzos Georgios

Tsivgoulis Georgios

Filho Geraldo Lorenzi

Salazar Gerard

Bosco Gerardo

Kuiper Gerhardus

Mintziori Gesthimani

Colussi Giacomo

Pucci Giacomo

Zoppini Giacomo

Juli Giada

Lavranos Giagkos

Girolomoni Giampiero

D’Andrea Giancarlo

Marenzi Giancarlo

Caiazzo Gianluca

Iacobellis Gianluca

Serafini Gianluca

Balzano Gianpaolo

Zerbini Gianpaolo

Serrancoli Gil

Corazza Gino Roberto

Alves Giordano

Barbanti Brodano Giovanni

Dapri Giovanni

Di Zenzo Giovanni

Guaraldi Giovanni

Serio Giovanni

Patel Girijesh

Bhatt Girish

Zanca Gisele

Fiorini Giulia

Cavestro Giulia Martina

Tocci Giuliano

Andò Giuseppe

Biondi-Zoccai Giuseppe

Ceraudo Giuseppe

Di Costanzo Giuseppe

Di Lorenzo Giuseppe

Gargiulo Giuseppe

Lepore Giuseppe

Lucarelli Giuseppe

Mulè Giuseppe

Procopio Giuseppe

Romito Giuseppe

Seghieri Giuseppe

Annicchiarico Giuseppina

Omura Go

Barbakadze Gocha

Kato Gohei

Oner Gokalp

Cuce Gokhan

Ji Gong-Jun

Pullas Gonzalo

Kaliappan Gopal

Hauser Goran

Poropat Goran

Angelique Gougelet

Alarcón Graciela

Louie Grant

Niro Grazia

Corbi Graziamaria

Antonino Graziano

Atkinson Greg

Hutter Gregor

Rodríguez-Boto Gregorio

Robbins Gregory

Taylor Gregory

Weitsman Gregory

D’Ascoli Greta Luana

Leontiadis Grigorios

Hao Guang

Wang Guanghong

Chen Guangming

Chen Guangming

Cai Guangyan

Zhang Guanyi

Ying Gui-Shuang

Zhang Guilian

Fond Guillaume

Piessen Guillaume

Herrera Guillermo

Zhang Guiming

Ji Guiyuan

Genc Gulsum

Lee Gun Woo

Loske Gunnar

Chen Guo

Zheng Guo-Qing

Li Guoan

Huang Guofu

Shan Guogen

Deng Guohong

Li Guojun

Zhou Guoying

Moreira Gustavo

Oliveira Gustavo

Sevincer Guzin

Jong Gwo-Ping

Farkas Jr. Gyula

Esendağlı Güneş

Ren H.

Park Ha Na

Salameh Habeeb

Gedik Habip

Lee Haekyung

Bachmann Hagen

Wang Haibin

Yang Haichun

Xu Haifei

Xu Haineng

Chen Hairong

Xie Haiting

Xiao Haiyan

Gu Haiyong

Liu Haizhou

Liu Haizhou

Hasegawa Hajime

Iizuka Haku

Batur Çağlayan Hale

Thayele Purayil Hamsa

Sheng Han

Wang Han

Cheng Han-Yi

Azzam Hanan

Guan Hanfeng

Schoellhammer Hans

Chen Hao

Deng Hao

Guo Hao

Liu Hao

Chen Haoyu

Potu Harish

Singh Harkirat

Burke Harry

Sugimura Haruhiko

Nakano Haruki

Yuksel Harun

Yazici Hasan

Zubair Haseeb

Heinzow Hauke

Gershengorn Hayley

Kalashyan Hayrapet

Tian He

Li Hecheng

Cho Hee Young

Joo Heesoo

Huang Hefeng

Ford Heide

Haller Heidemarie

Jenkins Helen

Maestrini Heloísa

Zhu Heming

Nienhüser Henrik

Wilson Henry

Tanowitz Herbert

Zhang Heye

Zhang Heye

Hamasaki Hidetaka

Yabuuchi Hidetake

Lazarus Hillard

Taki Hirofumi

Sone Hirohito

Yamashita Hiroko

Arai Hiromasa

Kondo Hiroshi

Nishida Hiroshi

Tanaka Hiroshi

Yatsuhashi Hiroshi

Aihara Hiroyuki

Isayama Hiroyuki

Matsumoto Hisako

Adachi Hisashi

Oshiro Hisashi

Minami Hitomi

Yokoyama Hitoshi

Lee Ho-Won

Husi Holger

Chang Hong

Li Hong

Weng Hong

Zhang Hong

Zhao Hong

Tan Hong Yien

Ren Hong-Gang

Tie Hong-Tao

Wang Honglin

Dong Hongmei

Zeng Hongwu

Zhao Hongxin

Zhang Hongxing

Guo Hongyan

Choi Hoon Young

Liang Houjie

Morris Howard

Yu Hsien-Chung

Chen Hsing-Yu

Shen Hsiu-Nien

Chang Hsueh-Wei

Han-Hwa Hu

Chen Hua

Cheng Hua

He Hua

Li Hua

Ling Hua

Liu Hua

Pang Hua

Tan Hua

Zhou Huacheng

Zhang Huafeng

Xu Huajun

Tu Huakang

Zhang Huan

Yu Huang-Ping

Liu Huayang

Chen Huey-Yi

Lin Hugo

Soudeyns Hugo

Dai Hui

Guo Hui

Huang Hui

Li Hui

Peng Hui

Zhang Hui

Tsai Hui-Ji

Hsieh Hui-Shan

Cao Huijuan

Wei Huiling

Tao Huiren

Khan Humaira

Gonzalez Humberto

Yang Hung-Chih

Huang Hung-Sheng

Kao Hung-Wen

Chiou Hung-Yi

Cao Huojun

Besiroglu Huseyin

Buyukgol Huseyin

Elsiesy Hussien

Lee Hyangsook

Lee Hye Won

Kang Hyeun-Ah

Kim Hyoun-Ah

Chung Hyun Chul

Shin Hyun-Woo

Kim Hyungjoon

Guimarães Hélio

Bauer Hélène

Lee I-Cheng

Hsin I-Fang

Huang I-Fei

Rissanen I.

Wallace Ian

Batal Ibrahim

Guler Ibrahim

Hatemi Ibrahim

Kilinc Ibrahim

Oda Ichiro

Tatsuno Ichiro

Parveen Iffat

Piras Ignazio

Okpechi Ikechi

Mokoagow Ikhsan

Yaylim Ilhan

Coven Ilker

Dastan Ilker

Gul Ilker

Fawzy Iman

Cho In Rae

Chattopadhyay Indranil

Bersch Ines

Tseng Ing-Jy

Koenigs Ingo

Arijs Ingrid

Heuch Ingrid

Waisman Ingrid

Yino Inomata

Jang Insoo

Jung Ioan

Bakolis Ioannis

Gkegkes Ioannis

Pliakos Ioannis

Tsougos Ioannis

Androulakis Ioannis I

Nabipour Iraj

Brčić Karačonji Irena

Hrstić Irena

Frachon Irene

Mukui Irene

Inan Irfan

Estevan Isaac

Gomez-Hurtado Isabel

Sada-Ovalle Isabel

Cleynen Isabelle

Sen Ishita

Arslan Ismail

Labgaa Ismail

Maatouk Ismaël

Alvarez-Twose Ivan

Vujkovic-Cvijin Ivan

Cruz Ivana

Passos Ives

Garcia De Gurtubay Iñaki

Flack J M

Mcneil J. Chase

Sokol Ja

Holmes Jacinta

Buhrow Jack

Ecanow Jacob

Sellarés Jacobo

Van Wouwe Jacobus P

Lenders Jacques

Pasternak Jacyr

Lee Jae Gil

Eo Jae Seon

Choi Jae-Mun

Koo Jaehyung

Bayry Jagadeesh

Kim Jaihwan

Howard James

Kellam James

Tabibian James

Tonsgard James

Willig James

Blaha Jan

Larmann Jan

Van Lunzen Jan

Cohen Tervaert Jan Willem

Kellett Jane

Vazquez-Mellado Janitzia

Wang Jann-Tay

Berecki-Gisolf Janneke

Minarovits Janos

Klaus-Peter Janssen

Seah Jarrel

Luke Jason

Springer Jason

Vanatta Jason

Wachsmann Jason

Yam Jason

Singh Jaspal

Wu Jau-Ching

Kojuri Javad

Yakoob Javed

Arpa Javier

De La Fuente Aguado Javier

Juega-Mariño Javier

Molina-García Javier

Ruiz-Martinez Javier

Bilal Jawad

Luther Jay

Siak Jay

Asirvatham Jaya

Krishnan Jayalakshmi

Hassan Jaythoon

Yombi JC

Bigna Jean Joel

Bouaziz Jean-David

Vincent Jean-Louis

Raoul Jean-Luc

Fermand Jean-Paul

Ackman Jeanne

Lechner-Scott Jeannette

Huang Jee-Fu

Chang Jeffrey

Knauf Jeffrey

Meyer Jeffrey

Staab Jeffrey

Jones Jeffrey A

Walline Jeffrey J

Celutkiene Jelena

Lee Jen-Kuang

Rychert Jenna

Berumen Jennifer

Duchon Jennifer

Fillaus Jennifer

Koplin Jennifer

Shum Jennifer

Mcgowan Jennifer A.

Klotsche Jens

Song Jeong Eun

Lee Jeong-Hoon

Adler Jeremy

Kers Jesper

Porta-Etessam Jesús

Sankar Jhuma

Heo Ji Haeng

Ryu Ji Kon

Chen Ji-Yih

Cao Jia

Fan Jia

Foo Jia Nee

Zhao Jia-Guo

Dong Jia-Hong

Choi Jiae

Geng Jiafeng

Chen Jiajia

Chen Jiajian

Chen Jiamin

An Jian

Chen Jian

Liu Jian

Wu Jian

Zhong Jian

Gao Jian Dong

Hu Jian-Kun

Ren Jianan

Cheng Jianding

Wu Jiang

Shen Jiangang

Fan Jiangao

Li Jiangfa

Wu Jianguo

Mao Jianhua

Wang Jianhui

Liu Jianping

Yin Jianyi

Wu Jianyong

Hao Jianyu

Shen Jianzhen

Yang Jiao

Hong Jiaxu

Li Jie

Liu Jie

Meng Jie

Shen Jie

Tang Jie

Tao Jie

Xiang Jie

Xu Jiejie

Kothari Jignesh

Zhang Jiming

Efird Jimmy

Di Jin

He Jin

Sheng Jin

Xu Jin

Yang Jin

Zhao Jin

Zhao Jin

Paeng Jin Chul

Jeon Jin Pyeong

Shih Jin-Chung

Xu Jin-Fu

Hong Jin-Liern

Wu Jin-Ming

Suh Jin-Suck

Yan Jinchuan

Zheng Jine

Bhagat Jinendra

Qu Jinfeng

He Jing

Su Jing

Wu Jing

Xie Jing

Xie Jing

Xu Jing

Yi Jing

Zhang Jing

Zhao Jing

Liu Jingchun

Liu Jinghua

Li Jingmei

Tong Jingshan

Li Jingting

Wang Jingyu

Yang Jingyun

Wang Jinheng

Yue Jinhuan

Zhang Jinqiang

Li Jinrong

Xu Jinrui

Liu Jinxu

Deng Jiong

Hu Jiong

Wu Jiong

Xu Jiru

Zhao Jiuliang

Wang Jiun-Ling

Chiang Jiun-Yang

Zhang Jiuquan

Tan Jiwei

Gu Jk

Ramos Jm

Ferreira Joana

Wardlaw Joanna

Lind Joanne

Oliver Joanne

Oliveira-Costa Joao Paulo

Schneider Jochen

Wedemeyer Jochen

Fink Jodok

Rule Jody A

Falkinham Joe

Aronowitz Joel

Dréanic Johann

Escher Johanna

Temime Johanna

Mayr Johannes

Dimopoulos Johannes C. Athanasios

Chambers John

Collins John

Hammond John

Hsiang John

Nance John

Santoshi John

Thompson John

Zaunders John

Norvell John Paul

Padulo Johnny

Ranstam Jonas

Anolik Jonathan

Loree Jonathan

Myers Jonathan

Wong Jonathan

Kim Jong Hae

Hyun Jong Jin

Choi Jong Won

Kim Jong-In

Lee Jongmin

Cho Joo Young

Choi Joon Young

Kleeff Jorg

Lizandra Jorge

Lemos Jose

Pacheco-Nunez Jose

Sandoval Jose

Valbuena Jose

Priego Quesada Jose Ignacio

Bermejo Jose Luis

Miro Jose M

Oliveira Jose Mario Franco

Michl Josef

Llibre Josep M

Ribera Josep-Maria

Brugada Joseph

Larmarange Joseph

Morales José

Esteves Veiga José Carlos

Macedo José Fernando

Ooi Jot Hui

Evangelista Joy

Ruaro João

Sabino João

Wang Ju

Yang Ju

Yang Ju Dong

Caicedo Juan

Camacho Juan

Ding Juan

Galvez Juan

Serna Juan

Zhao Juan

Zou Juan

Gonçalves Júnior Jucier

Wang Jue

Lin Jui Hsiang

Hsu Jui-Ting

Karakoumis Julia

Samuelson Julia

Smith Gagen Julie

Maitre Julien

Pottecher Julien

Hou Juliet

Alvarez-Pitti Julio

Collazos Julio

Hernandez Julio

Blanco Julià

Benito-León Julián

Chen Jun

Du Jun

Jin Jun

Li Jun

Li Jun

Li Jun

Li Jun

Long Jun

Ma Jun

Niu Jun

Pu Jun

Shi Jun

Wang Jun

Zhao Jun

Zhou Jun

Choi Jun Yong

Do Jun Young

Dong Junchao

Jin Junfei

Lee Jung Hwan

Lai Jung-Nien

Ishioka Junichiro

Sageshima Junichiro

Liu Junjie

Ye Junna

Wen Junping

Fraser Justin

Keogh Justin

Lefèvre Jérémie

Schattenberg Jörn

Scherberich Jürgen

Krause K

Deng Kai

Wermker Kai

Chang Kai-Po

Liu Kai-Xiong

Sun Kaiqi

Liang Kaiwei

Cao Kaixiang

Liu Kaiyong

Bhamidimarri Kalyan Ram

Cheng Kam Chau

Le Kang

Wang Kang

Khandelwal Kanika

Narain Kanwar

Tandon Kanwarpreet

Soni Kapil Dev

Khow Kareeann

Neemann Kari

Leander Karin

Pillunat Karin

Olsen Karina

Horisberger Karoline

Stolarz-Skrzypek Katarzyna

Parikh Kathan

Staufer Katharina

Baquerizo Nole Katherine

Peters Katherine

Kieran Kathleen

Hagberg Katrina

Iijima Katsunori

Tripathi Kaushlendra

Alquadan Kawther

Khaw Kay-Tee

Arima Kazuhiko

Matsumoto Kazumasa

Chang Ke-Vin

Ding Kefeng

Zhou Kehua

Ikeda Kei

Takayama Kei

Suzuki Keisuke

Zandi Keivan

Wong Kelvin

Özen Kemal Emre

Chavin Ken

Shih Kendrick

Yoshimitsu Kengo

Imai Kenichiro

Tsuchida Kenji

Cardona Kenneth

Inamura Kentaro

Miyai Kentaro

Sakamaki Kentaro

Singh Kern

Wu Kerui

Jean Kevin

Abu Ali Khaled

Saad Khaled

Tafran Khaled

Al-Rubeaan Khalid

Sung Ki Chul

Smith-Whitley Kim

Huynh Kim Hieu

Arimura Kimiyoshi

Chiu King-Wah

Batra Kiran

Talekar Kiran

Jordan Kirk

Bakrania Kishan

Suzuma Kiyoshi

Giannoulis Kleanthis

Janssen Koen

Abe Koichiro

Wu Kongming

Griva Konstadina

Bachoumas Konstantinos

Dafopoulos Konstantinos

Dimas Konstantinos

Dimas Konstantinos

Kamposioras Konstantinos

Papamichail Konstantinos

Tsilidis Konstantinos

Tziomalos Konstantinos

Jacob Korula

Perisinakis Kostas

Azuma Kosuke

Hashimoto Kosuke

Tamura Kouichi

Tummala Krishna

Ikenberg Kristian

Andresen Kristoffer

Allan Krumholz

Bojakowski Krzysztof

Szmyt Krzysztof

Yu Kuang-Hui

Liao Kuang-Ming

Ptaszkowski Kuba

Lee Kuei-Chuan

Kim Kun Hyung

Chan Kun-Ming

Chou Kun-Ta

Bai Kunhao

Huang Kuo-Chin

Chen Kuo-Hu

Ruetzler Kurt

Chan Kwan-Leung

Wang Kwua-Yun

Lee Kyong Joo

Jung Kyu Sik

Atzori L

Yessayan L

Boyer L.

Straney Lahn

Ren Laibin

Sherief Laila

Kang Laiyi

Anand Lakesh

Krishnakumar Lakshmi

Yaddanapudi Lakshmi Narayana

Lucy Lamb

Assoumou Lambert

Gao Lan

Xiao Lanbo

Zhang Lanjing

Breimer Lars

Donath Lars

Svendsen Lars Bo

Bonanni Laura

Boomer Laura

Orsolini Laura

Alexander Lauren

Fishbein Lauren.

Auboire Laurent

Boyer Laurent

Garderet Laurent

Wen Lebin

Chmielik Lechoslaw

Hebert Lee

Chuang Lee-Ming

Dong Lei

Liu Lei

Wang Lei

Zhang Lei

Zhang Lei

Sun Leimin

Wang Leiming

Maggiori Leon

Sowah Leonard

Szydlowski Leslaw

Hassell Lewis

Du Li

Jiang Li

Lai Li

Xiaoyan Li

Yong Li

Zhang Li

Lee Li-Ang

Sung Li-Chin

Chen Li-Da

Cui Li-Gang

Hsin Li-Jen

Huang Li-Kai

Hung Li-Wei

Lin Lian-Yu

Wei Liang

Xu Liang

Mu Liangshan

Xu Lichen

Li Lieber Po-Hung

Lin Lifeng

Duan Lihua

Xu Lihua

Cheng Lijun

Liu Lijun

Xie Lijun

Chen Lili

Qin Lili

Wang Lili

Rogozea Liliana

Li Liliang

Zhang Liman

Chen Limin

Li Liming

Chen Lin

Fan Lin

Jin Lin

Li Lin

Wang Lin

Su Lin-Hui

Gao Linbo

Lu Linchao

Macheka Linda

Keir Lindsay

Zeng Ling-Li

Zheng Linghua

Meng Linghui

Liao Lingjie

Wang Lingling

Yang Lingyao

Long Lingyun

Lazar Liora

Schimmenti Lisa

Vanhouwelingen Lisa

Yueju Liu

Wang Liuyang

Hegerova Livia

Lumbroso-Le Rouic Livia

Ding Liya

Cui Liyang

Xie Liyi

Lin Lizhou

Andersen Ljubica

Znaor Ljubo

Brewster Lm

Spector Logan

Bhushan Lokesh

Sunde Lone

Jiang Long

Fan Longchang

Melit Lorena Elena

Orci Lorenzo

Spineli Loukia Maria

Hubers Lowiek

Cao Lu

Huang Lu

Lu Lu

Yao Lu

Zhang Lu

Tong Lu-Sha

De Haro Luc

De Santo Luca

Faloppi Luca

Frulloni Luca

Valenti Luca

Vigano Luca

Zhu Lucheng

Campos Luciana Aparecida

Delgado-Plasencia Luciano

Drager Luciano

Quaranta Luciano

Tarantino Luciano

Conterno Lucieni

Mercadal Lucile

Boglione Lucio

Chen Lucy

Goulding Lucy

Abenavoli Ludovico

Di Serafino Luigi

Marano Luigi

Carvajal Carmona Luis

Gonzalez Luis

Lopes Luis

Medina Andrade Luis

Rato Luis

Rodrigo Luis

Taborda-Barata Luis

González Naranjo Luis Alonso

Castillo Luis Manuel

Oliveira Luis Vicente Franco

Lima Luiz

Pimenta Luiz

Guilherme Luiza

Iselin Lukas

Grochola Lukasz

Szarpak Lukasz

Moore Luke

Chan Lung

Eccersley Lydia

Demir Lütfi

Mavros M

Piga M

Rodriguez Esteban M

Rajan M G R

Jacob M J

Bernal-Lopez M Rosa

Foppa M.

Gil-Alegre M. Esther

Zhanlong Ma

Vancamelbeke Maaike

Banach Maciej

Durand Madeleine

Xu Mafei

Kotb Magd

Martinez-Tome Magdalena

Fallah Mahdi

Haghighatafshar Mahdi

Padanad Mahesh S

Rafieian-Kopaei Mahmoud

Arabul Mahmut

Dey Mahua

Taylor Maida

Nowakowski Maja

Sabol Maja

Moshirfar Majid

Lemyze Malcolm

Lee Malrey

Mochizuki Manabu

Wong Mandy

N Manikandan

Gaddh Manila

Rocha Manoel

Kumar Manoj

Sarma Manoj

Wang Manqi

Lemos Manuel

Mao Mao

Fischler Marc

Leone Marc

Singer Marc

Vorpahl Marc

Fischer Marc-Olivier

Nebbioso Marcella

Dougan Marcelle

Fe Marchlinski

M.M. Pizzichini Marcia

Garrison Dytz Marcio

Kiuchi Marcio

Alves Marco

Bueter Marco

Carotenuto Marco

Cascella Marco

Falcone Marco

Fichera Marco

Pinho Marco

Solmi Marco

Vieira Guedes Marco

De Carvalho-Filho Marco Antonio

Hartleb Marek

Chen Margaret

Gavriatopoulou Maria

Gazouli Maria

Ruffino Maria

Tolia Maria

Mussi Maria Carolina

Cintra Maria Leticia

Dore Maria Pina

Zegpi Maria Soledad

Monteiro Mariana

Alesi Marianna

Gunell Marianne

Preau Marie

Rousset Marie

Iliou Marie-Christine

Landsmeer Marieke L.A.

Cerquetti Marina

Prado Marina

Cazzola Mario

D’Elios Mario

D’Elios Mario

Giuffrè Mario

Couch Marion

Tasoulis Marios-Konstantinos

Adamek Mariusz

Dąbrowski Mariusz

Birkenbach Mark

Bradov Mark

Cookson Mark

Haas Mark

Kaddumukasa Mark

Logue Mark

Zelić Marko

Diener Markus

Mohaupt Markus

Schneider Markus

Dąbrowska Marta

Trendelenburg Marten

De Borst Martin

Floer Martin

Hubner Martin

Lhuaire Martin

Montenovo Martin

Risch Martin

Wagner Martin

Bukhari Marwan

Vilay Mary

Zhao Mary

Sánchez-García María Esther

Pérez Elías María Jesús

Pérez Elías María Jesús

Couce María-Luz

Yoshimura Masafumi

Jinzaki Masahiro

Hara Masaki

Omae Masami

Mario Masarone

Takeuchi Masaru

Ohsawa Masato

Sepehrimanesh Masood

Berretta Massimiliano

Bissolati Massimiliano

Pau Massimililano

Fusconi Massimo

Pasquini Massimo

Tusconi Massimo

Hidvegi Mate

Wiest Matheus

Sewell Mathew

Baumert Mathias

Bähr Mathias

Poussel Mathias

Fiore Mathieu

Ferro Matteo

Pacini Matteo

Brothers Matthew

Cohn Matthew

Halanski Matthew

Kircher Matthew

Pincus Matthew

Prof. Cook Matthew

Roberts Matthew

Silva Matthew

Buechter Matthias

Kreppel Matthias

Komorowski Matthieu

Brouwer Matthijs

Mandorfer Mattias

Lemoine Maud

Battaglia Parodi Maurizio

Iacobone Maurizio

Russello Maurizio

Oliveira Mauro

Podda Mauro

Eric Maury

Tan Maw Pin

Dieterich Max

Battistella Maxime

Tran Maxine

Rodriguez-Violante Mayela

Ramanathan Meenakshi

Crane Megan

Kannan Meganathan

Caulfield Meghan

Namdar Mehdi

Şentürk Mehmet

Artaç Mehmet

Balioglu Mehmet

Erdoğan Mehmet

Sonmez Mehmet

Turan Mehmet

Turkcu Mehmet

Ugurlu Mehmet

Baktir Mehmet Akif

Dundar Mehmet Akif

Sözen Mehmet Enes

Yazar Mehmet Fatih

Yilmaz Mehmet Tugrul

Chung Mei

Shi Mei

Song Mei

Zhang Mei

Wu Mei-Yi

Yu Meifang

Wang Meilin

Lu Meixia

Fang Meiyu

Horter Melanie

Price Melissa

Teo Melissa

Zhang Melvyn

Lee Meng

Liu Meng

Xu Meng

Tsai Meng-Han

Dai Menghua

Wang Mengjie

Chen Mengshi

Li Mengtao

Falip Mercé

Aydin Mesut

Ayer Mesut

Ogrendik Mesut

Gündüz Metin

Hambraeus Mette

Kim Mi Na

Liu Miao

Teng Miao

Yu Miao

Kukla Michał

Cripps Michael

Frass Michael

Glocker Michael

Grant Michael

Green Michael

Guzman Michael

Link Michael

Liu Michael

Nurmohamed Michael

Paech Michael

Scholl Michael

Simon Michael

Soussan Michael

Taylor Michael

Kostapanos Michael S.

Barak Michal

Drancourt Michel

Gonzalez Michel

Ammendola Michele

Ciccarelli Michele

Coppola Michele

Figus Michele

Senni Michele

Torella Michele

Pillat Micheli

Kim Michelle

Yip-Schneider Michelle

Fukui Michiaki

Nakayama Michihiro

Satoh Michihiro

Camafort-Babkowski Miguel

Gonzalez-Gay Miguel

Gorgolas Miguel

Pérez-Fontán Miguel

Sathekge Mike

Petranovic Milena

Pesic Milica

Tavella Mimmo

Chen Min

Chen Min

Liu Min

Tan Min

Xu Min

Zong Min

Kim Min Jung

Mohareb Mina

Psichogiou Mina

Suh Mina

Yu Minbin

Li Ming

Lin Ming Kai

Hou Ming-Chih

Yu Ming-Lung

Lai Ming-Wei

Hsiao Ming-Yen

Zhang Minggang

Zheng Minghua

An Mingrui

Zhao Mingrui

Okubo Minoru

Weinberger Miriam

Sotto Mirian

Simunic Miroslav

Milic Mirta

Koz Mitat

Isobe Mitsuaki

Shimoda Mitsugi

Kawano Mitsuhiro

Knotek Mladen

Petrovečki Mladen

Amiri Mohamadreza

Abdalaal Mohamed

Alboraie Mohamed

Arruj Mohamed

Bakri Mohamed

El Kassas Mohamed

El Sorogy Mohamed

El-Nady Mohamed

Fadl Elmula Mohamed

Khalil Mohamed

Mekky Mohamed

Wifi Mohamed

Derakhshan Mohammad

Khan Mohammad Aslam

Fallahzadeh Mohammad Kazem

Tahoori Mohammad Taher

Elsheemy Mohammed

El-Badawy Mohja

Hamidpour Mohsen

Elalfy Mohssen

Kupolati Mojisola D.

Boaz Mona

Wang Mongheng

Hasnat Monika

Sherman Morris

Breindahl Morten

Sircar Mrinal

Yang Mu

Cheng Mu-Hua

Asif Muhammad

Kamal Muhammad

Makusidi Muhammad

Khan Muhammad Ali

Mubarak Muhammed

Sahin Muhammed

Jamal Muhsin

Calikoglu Mukadder

Agrawal Mukta

Cao Muqing

Malligaraman Murali

Kondapaneni Muralidhar

Cakir Murat

Harputluoglu Murat

Yuksel Murat

Barbhuiya Mustafa

Gursoy Mustafa

Keles Mustafa

Rasid Toksoz Mustafa

Lee Myeong Soo

Sparo Mónica

Danisman N

Gökbayrak N Simay

Benito N.

Cho Na-Eun

Mukherjee Nabanita

Yang Nachen

Alatrakchi Nadia

Salerno Nadia

Klafke Nadja

Anreddy Nagaraju

Issa Naim

Solanki Naimesh

Solanki Naimesh

Muto Nair

Ku Nam Su

Kim Nam-Kyu

Liu Nan

Berliner Nancy

Narayanan Nandakumar

Mani Nandini

Pandit Nandini

Yashimura Naoki

Palla Narayan

Joshi Narendra

Nair Narendra

Holmes Natasha E.

Vikram Naval

Win Nay

Kyla Naylor

Dhaun Neeraj

Saraf Neeraj

Merrett Neil

Mahdieh Nejat

Gouveia Nelia

Astur Nelson

Alberto Neville

Wang Niansong

Arzouni Nibal

Sculthorpe Nicholas

Gebruers Nick

Steckhan Nico

Palestini Nicola

Squillace Nicola

Veronese Nicola

Girerd Nicolas

Goossens Nicolas

Javaud Nicolas

Leveziel Nicolas

Noel Nicolas

Poté Nicolas

Bernard Nicole

Ronda Nicoletta

Zimmermann Nicolle

Biswas Nidhan K.

Wiinberg Niels

Daoud Nihaya

Tyagi Nikhil

Anagnostou Nikolaos

Chatzizacharias Nikolaos

Tsoukalas Nikolaos

Eleftheriadis Nikolas

Pantazis Nikos

Talele Nilesh

Penha-Silva Nilson

Skjæret Maroni Nina

Ge Ning

Li Ning

Li Ningdong

Kumar Nitasha

Gupta Nitin

Matsunaga Nobuaki

Toshikuni Nobuyuki

Manabe Nodoka

Brueggemann Norbert

Hynes Noreen

Mostafa Norhan

Ojeda Norma

Galbreith Norman

Fernández-Hidalgo Nuria

Homaira Nusrat

Groenewold Nynke

Joo Hyun O

Yaldizli O

Pecurariu Oana

Săndulescu Oana

Cohen Ohad

Hussein Okasha

Uthman Olalekan

Parssinen Olavi

Munhoz Leite Olavo

Nielsen Ole Haagen

Mozenska Olga

Simionescu Olga

Galasso Olimpio

Hunsicker Oliver

Schildgen Oliver

Strobel Oliver

Gié Olivier

Huttin Olivier

Ayodele Olugbenga

Ameh Oluwatoyin

Uysal Omer

Fiala Ondrej

Celik Onur

Panagiotou Orestis

Binici Orhan

Özdiler Orhan

Nomura Osamu

Satoshi Osawa

Evbuomwan Osayande

Cekic Osman

Liu Ou

Salim Ozan

Tabatabaei-Malazy Ozra

Jemenez-Fonseca P

M’Bappé P

Ferrer Pablo

Irimia Pablo

Uribe Pablo

Manoharan Palanikumar

Leal Pamela

Sass Pamela

Athanasopoulos Panagiotis

Korovessis Panagiotis

Vasilakis Panagiotis

Karaiskos Pantelis

Di Carlo Paola

Queirolo Paola

Arcidiacono Paolo

Brusini Paolo

Fiorina Paolo

Fogagnolo Paolo

Gisondi Paolo

Salvi Paolo

Schena Paolo

Parekh Parag

Badiee Parisa

Tabrizian Parissa

Mohan Parul

Probst Pascal

De Negri Pasquale

Striano Pasquale

Silsupadol Patima

Carreira Patricia

Jimenez Patricia

Nielsen Patricia

Polanco Patricio

Sequeira-Byron Patrick

Gabikian Patrik

Proia Patrizia

Van Daele Paul

Peixe Paula

Ramírez Paula

Criado Paulo

Dourado Paulo

Ocke Reis Paulo

Braz-Silva Paulo Henrique

Chernyavskiy Pavel

Malindretos Pavlos

Msaouel Pavlos

Nachulewicz Paweł

Gondim Teixeira Pedro Augusto

Chen Pei-Lung

Wang Peiji

Haddad Peiman

Song Peipei

Shou Peishun

Murangandi Pelagia

Horowitz Peleg

Musto Pellegrino

Liao Pen-Chih

Du Peng

Liu Peng

Xu Peng

You Fan Peng

You Fan Peng

Yuan Peng

Yu Pengfei

Yang Penghua

Wan Pengxia

Qian Pengxu

Dousdampanis Periklis

Boor Peter

Daley Peter

Harri Peter

Havens Peter

Schemmer Peter

Sykora Peter

Van Der Wurff Peter

Warnke Peter

Whittaker Peter

Nilsson Peter M

Kanovsky Petr

Klose Petra

Bjornstad Petter

Holland Philip

Müller Philip

Omotosho Philip

Seo Philip

Sand Philipp

Stawowy Philipp

Zanger Philipp

Domenech Philippe

Sinxadi Phumla

Neri Piergiorgio

Allemann Pierre

Filhine-Tresarrieu Pierre

Fournier Pierre

Guinot Pierre Gregoire

Depuydt Pieter

Canetta Pietro

Serra-Añó Pilar

Cheng Pin-Nan

Li Ping

Ren Pingping

Kozlowski Piotr

Wegrzyn Piotr

Maglione Pj

Cheng Po-Ching

Huang Po-Yen

Hsu Pokuei

Shantavasinkul Prapimporn

Joshi Prathamesh

Chowdhury Pratim

Rawat Pratima

Carrera Pricivel

James Prince

Oliveira Priscila

Trindade Priscila

Callahan-Lyon Priscilla

Chidambaram Priyadarshini

Shah Priyank

Verma Priyanka

Talbot Prue

Zdziarski Przemyslaw

Cheung Pui Yee

Bagga Puneet

Bagga Puneet

Lin Pyng-Jing

Kelemen Péter Bertalan

Gong Qi

Guo Qi

Zhang Qi

Liu Qi-Fa

Wu Qi-Jun

Wang Qian

Jie Qiang

Li Qiang

She Qiang

Su Qiang

Yan Qiang

Mao Qianguo

Ma Qianyi

Wan Qiaoqiao

Qi Qibin

Ding Qiliang

Chen Qing

Wang Qing

Yao Qing

Zhao Qinglan

Ni Qingqiang

Hou Qingtao

Song Qingxuan

Zhang Qinhong

Wang Qiong

Wang Qiongxin

Wang Quan

Wang Quan

Li Quanlin

Gopen Quinton

Ciampi Quirino

Sun Qun

Beurskens R

Jasmin R

Shivaprakash R

Juan R. San

Gupta Raavi

Edwards Rachael

Evans Rachel

Luciano Rafael

López-Andújar Rafael

Badolato Raffaele

De Palma Raffaele

Pezzilli Raffaele

Pulli Raffaele

Franciotti Raffaella

Al-Dulaimi Ragheed

Khosla Rahul

Parghane Rahul

Chaudhary Rahul K

Tarun Rai

Rasiah Rajah

Nagarajan Rajakumar

Kanchanapally Rajashekhar

Balkrishnan Rajesh

Gupta Rajesh

Mohandas Rajesh

Thippeshappa Rajesh

Sarkar Rajiv

Jha Rajneesh

Rani Rajni

Kochhar Rakesh

Bauer Ralf

Debiasi Ralph

Rahme Ralph

Vignesh Ramachandran

Venugopalan Ramakrishna

Mani Ramakrishnan

Bhat Ramesh

Sudhagoni Ramu

Messiah Ramy

Dajani Rana

Hennigar Randolph

Sweis Randy

Sarkis Rani

Belzeaux Raoul

Favory Raphael

Hau Raphael

Burrows Raquel

Kanda Raquel

El Etreby Rasha

Chaudhary Rashmi

Tiwari Rasna

Samy Ravi

Kullar Ravina

Regatte Ravinder

Ankathil Ravindran

Liu Raymond

Rosales Raymond

Wong Raymond

Morfin-Otero Rayo

Desdicioglu Raziye

Multescu Razvan

Kanungo Reba

Deal Rebecca

Manno Rebecca

Aktimur Recep

Sade Recep

Hegazi Refaat

Williams Regan

Arita Reiko

Meester Reinier

Talhout Reinskje

Assis Renata

Pospischill Renata

Argirò Renato

Rodriguez Gutierrez Rene

Liu Rengyun

Lim Renly

Bao Renyue

Ratnawati Retty

Howden Reuben

Gandelman-Martin Revital

Lazaro Reynaldo

Elahimehr Reza

Nemati Reza

Mackey Rh

Cervera Ricard

Fontes-Carvalho Ricardo

Nahas Ricardo

Robles Ricardo

Romiti Ricardo

Forastiero Ricardo Raul

Autorino Riccardo

Giampieri Riccardo

Fagugli Riccardo Maria

Tyagi Richa

Do Richard

Doty Richard

Nelson Richard

Slart Riemer

Siciliano Rinaldo

Nakanishi Rine

Santos Rocha Rita

Romee Rizwan

Bailey Robert

Barkin Robert

Belvis Robert

Chen Robert

Scofield Robert

Speth Robert

Steiner Robert

Sundel Robert

Weiss Robert

De Rosa Roberta

Di Giacomo Roberta

Ficarelli Roberta

Pili Roberta

Priori Roberta

Vasconcelos Roberta

Di Donato Roberto

Gerli Roberto

Giacomelli Roberto

Pedrosa Roberto

Salvatori Roberto

Salvia Roberto

Troisi Roberto Ivan

Buerki Robin

Ho Robin Ht

Di Mascio Rocco

Kim Rock Bum

Nuñez-Miller Rodolfo

Canellas Rodrigo

Fraga-Silva Rodrigo

Jiménez-García Rodrigo

Rodrigues Rodrigo

Bennink Roelof

Leira Rogelio

Klotz Roger

Castilho Rogerio

Passos Rogerio

Khera Rohan

Mahabaleshwarkar Rohan

Sharma Rohini

Panchakshari Rohit

Hamam Rola N.

Jamora Roland Dominic

Leischik Roman

Shapiro Ron

Pelletier Ronald

Luo Rong

Yang Rongqiang

Lu Rongze

Sicari Rosa

Arcone Rosaria

Lisboa Rose

Olivero Rosemary

Di Trolio Rossella

Sá Rosália

Mccoy Rozalina G.

Wu Ruey-Meei (Robin)

Tian Rui

Wang Rui

Zhong Rui

Jia Rui-Peng

Li Ruifang

Deng Ruishu

Lin Ruiting

Fan Run

Hong Ruoxi

Sahasrabudhe Ruta

Sadikot Ruxana

Liu Ruya

Helmick Ryan

Merrell Ryan

Thomas Ryan

Yamada Ryotaro

Gawda Ryszard

Fuertes S

Parida S

Hsu S.-J.

Findik Siddika

Nseir Saad

Sorrentino Sabato

Anis Sabiha

Turdean Sabin

Bagaglio Sabrina

Gupta Sachin

Deshmukh Sachin Kumar

Sepanlou Sadaf

Nandwana Sadhna

Khan Sadia

Lee Sae Hwan

Alzghari Saeed

Liu Sai

Sankaralingam Saikolappan

Kazuyoshi Saito

Toro Sakuragi

Khaled Salama

Tamer Salem

Coskun Salih

Ribeiz Salma

De Rosa Salvatore

Longman-Mills Samantha

El-Ganainy Samar

Sheth Sameer A.

Shehata Sameh

Akbulut Sami

Gupta Md Samir

Parikh Samir

Chandramathi Samudi

Deshayes Samuel

Hohmann Samuel

Käser Samuel

Serbest Sancar

Dhall Sandeep

Julka Sandeep

Roth Sandor

Farkas Sanja

Singh Sanjay

Bhoi Sanjeev

Chawla Sanjeev

Kumar Sanjeev

Agarwal Sanjit

Vij Sanjiv

Garcia-Lopez Santiago

Perez Patrigeon Santiago

Nandigam Santosh

Yadav Santosh

Arévalo Sara

Awad Sara

Gandini Sara

Garcia-Ptacek Sara

Imanpour Sara

Massironi Sara

Ahmadi-Erber Sarah

Schmalzle Sarah

Edmondson Sarah-Jayne

Verstockt Sare

Tafelski Sascha

Handari Saskia

Shanbhag Satish

Barnawal Satish Prasad

Masuda Satohiro

Takahashi Satoru

Hoshide Satoshi

Inoue Satoshi

Alshryda Sattar

Pany Satyabrata

Chawla Saurabh

Caini Saverio

Ohn Mar Saw

Zyoud Sa’Ed H.

Go Se-Il

Polster Sean

Başyiğit Sebahat

Müller Sebastian

Leone Sebastiano

Kibaroğlu Seda

Gupta Seema

Azab Seham

Kiliç Selim

Cernea Selma

Kuyucu Semanur

Kang Seok Hui

Yang Seokjeong

Kim Seong Hoon

Ahn Seong Joon

An Seong Soo

Kwon Seong Young

Khodaverdi Sepideh

Shokouhi Sepideh

Duarte Sergio

Gonzalez Bombardiere Sergio

Kellesarian Sergio

Prieto-González Sergio

Erdogan Serpil

Altay Servet

Sankaran Sethuraman

Han Seung Hyeok

Lee Seung-Hwan

Mousavi Seyed

Kassaian Seyed Ebrahim

Yazar Seyhan

Günes Sezgin

Yi Sha

Bazmi Shabnam

Azim Shafquat

Aggarwal Shagun

Rashed Shah Manzur

Merchant Shaila

Kumar Shaji

Chen Shan

Wang Shan

Yu Shan

Xie Shang

Wang Shang-Yu

Zhao Shangang

Esaki Muthu Shankar

Flood-Nichols Shannon K.

Hawkins Shannon M.

Hsia Shao-Hsuan

Chuang Shao-Yuan

Tang Shaojun

Shen Shaomin

Su Shaoyong

Krishnamurthy Sharmitha

Tamir Sharon

Wu Shasha

Bagchi Shashwatee

Sen Shaundeep

Sukumaran Shawgi

Parker Shefton

A.M.K. Sheik Abdul Khadir

Rizzo Shemra

Chen Sheng

Li Sheng

Li Sheng

Song Sheng

Lin Sheng-Hsiang

Rao Sheng-Xiang

Wu Shengjie

Fan Shengjun

Huang Shengping

Li Sheyu

Shao Hua Shi

Li Shi-Jun

Guo Shicheng

Xu Shidong

Yang Shigao

Suzuki Shigeaki

Kotake Shigeru

Toyoda Shigeru

Chen Shih-Ann

Liu Shih-Feng

Hung Shih-Han

Sung Shih-Hsien

Chang Shih-Liang

Chang Shih-Lun

Huang Shih-Ting

Zhang Shijia

Liu Shijie

Zhao Shilin

Motohashi Shinichiro

Xie Shiqi

Feng Shiqing

Li Shiri

Yan Shirley

Fong Shirley S.M.

Sarin Shiv Kumar

Kaushik Shivani

Ishikawa Shizukiyo

Shoham Shmuel

Ganesan Shobana

Fukui Shoichi

Taghipour Zahir Shokouh

Elahi Shokrollah

Choudhury Shreyasi

Yamashita Shu-Ichi

Chao Shu-Ping

Jiang Shuai

Wang Shuaishuai

Guo Shuang

Tang Shuang

Xia Shuang

Lin Shuibin

Chang Shun-Jen

Qiao Shuxi

Pawar Shwetal

Bilasy Shymaa

Basaran Sibel

Kiran Sibel

Shao Sida

Tyagi Siddhartha

Muhlack Siegfried

Zhao Sihai

Liu Sijun

Alves Pereira Silvana

Bellando Randone Silvia

Miano Silvia

Misasi Silvia

Porcu Silvia

Kircher Simon

Panter Simon

Schneider Simon

Gurzu Simona

Gulletta Simone

Mocellin Simone

Ribero Simone

Genovesi Simonetta

Yuen Siu Tsan

Yedlapati Siva Harsha

Gupta SK

Diaconescu Smaranda

Sasindran Smitha

Kim So Ri

Manzoor Sobia

Dhaese Sofie

Midgley Sofie

Retamozo Soledad

Amornyotin Somchai

Dayama Sonal

Jin Song

Zhang Song

Santos-Lasaosa Sonia

Levanat Sonja

Steigen Sonja

Georgin-Lavialle Sophie

Tsiodras Sotirios

Singh Soudamani

Dasgupta Soumit

Cataland Spero

Manolakopoulos Spilios

Pagkratis Spyridon

Vallabhaneni Sreeram

Jaligama Sridhar

Velandy Sriram

Skevofilax Stamatios

Goldfarb Stanley

Hohaus Stefan

Schenk Stefan

Papatheodorou Stefania

Ballestri Stefano

Crippa Stefano

De Cillà Stefano

Rigattieri Stefano

Vujosevic Stela

Legriel Stephane

Birch Stephen

Grupke Stephen

Korsman Stephen

Mcsorley Stephen

Obaro Stephen

Reis Stephen

Rayhill Steve

Chmura Steven

De Decker Steven

Schwartz Steven

Polisner Stuart

Gaudry Stéphane

Zuily Stéphane

Raymond Stéphanie

Lee Su Jin

Park Su-Hyung

Nandakumar Subhadra

Vallabhapurapu Subrahmanya

Swaminathan Subramanian

Shukla Sudhanshu

Chavva Suhash

Su Sui-Lung

Chon Suk

Lee Sukmin

Bakanay Sule Mine

Demiryas Suleyman

Suman Suman

Ghosh Sumantra

Sundaraiya Sumati

Chhatre Sumedha

Huang Suming

Saha Sunandan

Veluswamy Sundar

Hong Sung Kyu

Hong Sung Kyu

Rao Sunil V

Singh Sunita

Kim Sunjung

Mahasirimongkol Surakameth

Sharma Surendra

Nair Suresh

Rana Surinder

Malcolm Smith Susan

Tsang Susanna

Djajalaksana Susanthy

Sharma Suvasini

Jain Suvi

Sousa Suzana

Ono Suzane

Lang Sven

Kjeldsen Sverre

Andrews Sweta

Moirangthem Sydney

Ohrt-Nissen Søren

Balamugesh T

Kim Tae Min

Gweon Tae-Geun

Kim Tae-Hun

Görgülü Tahsin

Sharon Tai Mei Ling

Tseng Tai-Chung

Yoshimoto Taichiro

Cheng Tain-Junn

Mushiroda Taisei

Kswahara Taishi

Arigami Takaaki

Horiuchi Takahiko

Okamoto Takahiro

Hiramitsu Takahisa

Yamaguchi Takamune

Dejima Takashi

Kawahara Takashi

Miura Takashi

Masui Takayuki

Kawaguchi Takehiko

Umemura Takeji

Tanos Tamara

Meyers Tammy

Mersich Tamás

Kraus Tanja

Mukherjee Tanushri

Desai Tanvi

Liu Tao

Shen Tao

Shen Tao

Sun Tao

Ye Tao

Shafi Tariq

Eghbal Eftekhaari Tasnim

Yoshida Tatsuya

Bharadwaj Tattwamasi

Soukup Tayana

Toptas Tayfur

Finseth Taylor

Wu Te-Chang

Belsuzarri Telmo Augusto

Wang Tengfei

Chen Tenghui

Bell Teresa

Cardoso Teresa

Mayordomo-Rodriguez Teresa

Michalczyk Teresa

Alraek Terje

Uprak Tevfik Kivilcim

Pham Thai

Field Thalia

Lytras Theodore

Kalampokas Theodoros

Emeto Theophilus

Herdeiro Theresa

Mesplede Thibault

Angelovich Thomas

Bice Thomas

Biedermann Thomas

Eigentler Thomas

Gary Thomas

Hocker Thomas

Kienbacher Thomas

Uray Thomas

Frieden Thomas R.

Gao Tian

Ma Tianle

Stankovic Tijana

Utkan Tijen

Farley Tim

Lahm Tim

Hu Tim Xiaoming

Karamitros Timokratis

Fitzgibbons Timothy

Moss Timothy

Stockwell Timothy

Chuang Ting-Wu

Xia Tingting

Tenenbaum Tobias

Jafari Koshki Tohid

Aksu Tolga

Hill Tom

Woodcock Tom

Horoch Tomasz

Akinyemiju Tomi

Ivankovic Tomislav

Chan Tommy

Ichikawa Tomoaki

Sonoo Tomohiro

Okada Tomohisa

Fujii Tomoko

Villén Tomás

Liu Tong

Lin Tonghui

Nilsson Torbjörn

Kudo Toshifumi

Shirai Toshiharu

Mayumi Toshihiko

Benamar Touria

Pilla Trinadha

Chao Tsu-Yi

Sadanaga Tsuneaki

Tsai Tsungyuan

Nishiyama Tsutomu

Yong Tuck Leong

Yong Tuck Yean

Sahutoglu Tuncay

Yu Tung-Min

Ji Tuo

Rantanen Tuomo

Karlidag Turgut

Lim Tze Peng

Shieh Tzong-Ming

Ho Tzong-Shiann

Boggi U

Ravnskov Uffe

Bilge Ugur

Nitsche Ulrich

Seybold Ulrich

Buchwald Ulrike

Kaiser Ulrike

Gauhar Umair

Thirugnanam Umapathi

Kalyoncu Umut

Das Undurti

Granacher Urs

Kiechl-Kohlendorfer Ursula

Govindarajulu Usha

Balaji Uthra

Reddy Uttam

Lambadiari Vaia

Da Silva Valdo José Dias

De Re Valli

Emani Vamsi

Giacomet Vania

Trachana Varvara

Lupu Vasile Valeriu

Giapros Vasileios

Berdoukas Vasili

Filiopoulos Vassilis

Alves Venancio

Garikipati Venkata

Krishnan Venkatesh

Abitbol Vered

Bachanova Veronika

Provencher Veronique

Kidir Veysel

Öner Veysi

Prado-Gasco Vicente

Falcó Vicenç

Kok Victor

Roberts Victor

Delgado Victoria

Menon Vidya

Velu Vijayakumar

Gopichandran Vijayaprasad

Maller Vijetha

Kotwal Vikram

Kašuba Vilena

Slev Vina

Mittal Vinay

Chung Vincent Ch

Panichi Vincenzo

Puro Vincenzo

Gupta Vineet

Nambudiri Vinod

Menon Vipin

Kumar Virendra

Brady Virginia

Kothari Vishal

Sharma Vishal

Yadav Vishal

Sankarasubramanian Vishwanath

Venketaraman Vishwanath

Vanar Vishwas

Di Lernia Vito

Martella Vito

Isaac Vivian

Kushnir Vladimir

Knesebeck Von Dem

Alkhiary Wael

Ansari Wahid

Al-Ghoul Walid

Ayoub Walid

Sifuentes-Giraldo Walter

Gathirua-Mwangi Wambui G.

Zhang Wan-Guang

Gu Wan-Jie

Lianghua Wang

Zheng Wang

David D. Weaver

Chen Wei

Chen Wei

Feng Wei

Gong Wei

Guo Wei

Huang Wei

Jiang Wei

Liao Wei

Qin Wei

Tu Wei

Wang Wei

Wu Wei

Xie Wei

Zhang Wei

Zhang Wei

Zhao Wei

Tsai Wei-Lun

Cai Weijia

Jiang Weijian

Wang Weilin

Hao Weimin

Chen Weina

Wang Weiqi

Chai Weiran

Lv Weiran

Meng Weixu

Martins Wellington

Jin Wen

Wu Wen

Yuan Wen

Zhou Wen

Yin Wen Yao

Yu Wen-Chung

Huang Wen-Hung

Wang Wen-Jun

Xiao Wen-Jun

Zhang Wen-Na

Guo Wenbin

Lei Wenbin

Lei Wenbin

Dahl Wendy

Liang Wenjie

Zhang Wenjie

Liao Wenjun

Yao Wenlong

Wang Wenshan

Tao Wensi

Li Wenting

Luo Wenting

Zhang Wenwen

Ding Wenyuan

Schöning Wenzel

Li Wenzhao

Liu Wenzhong

Angel Wes

Wojciechowska Wiktoria

Carey William

Clafshenkel William

Hu William

Lin William

Moore William

Wu William

Kohlhepp Willibald

Tam Wilson

Wysocki Wojciech

Chang Won Seok

Won Fen Wong

Kang Wonseok

Park Wooyeong

Ji Wu

Xie Wuxiang

García-Albéniz Xavier

García-Massó Xavier

Moisset Xavier

Muller Xavier

Zendjidjian Xavier

Li Xi

Qian Xi

Tan Xi

Wang Xi

Chen Xiabin

Wei Xian-Zhao

Chen Xiang

Gao Xiang

Gu Xiang

He Xiang

Hu Xiang

Shu Xiang

Wang Xiang

Wang Xiang

Zeng Xiang

Bu Xiangning

Zeng Xiangpei

Huang Xiangwei

Yang Xianrui

Zhang Xianwei

Zhou Xianxiao

Long Xiao

Zhang Xiao Dong

Lian Xiao-Feng

Chen Xiao-Yun

Yu Xiaobin

Zhu Xiaodong

Bian Xiaofang

Yang Xiaofang

Yu Xiaofei

Chu Xiaogang

Luo Xiaohe

Dai Xiaohua

Yu Xiaojia

Wang Xiaojing

Yue Xiaojing

Tan Xiaojun

Guo Xiaomao

Han Xiaomei

Li Xiaomin

Li Xiaoni

Chen Xiaoping

Qi Xiaoqiang

Yang Xiaoqin

Duan Xiaoqiong

Li Xiaoshan

Shi Xiaoshun

Zou Xiaowei

Rong Xiaoxiang

Zheng Xiaoxu

Lyu Xiaoxuan

Ding Xiaoyan

Zhang Xiaoyong

Hu Xiaoyu

Ming Xie

Chen Xin

Gao Xin

Jin Xin

Liu Xin

Liu Xin

Wang Xin

Xia Xin

Xia Xin

Chen Xin-Zu

Liu Xing

Zheng Xingnan

Diao Xingxing

Diao Xingxing

Li Xingyao

Zhang Xinhua

Jiang Xinyi

Jia Xinying

Weng Xisheng

Guo Xiuhua

Chen Xiukai

Mo Xiulei

Teitsma XM

Liu Xu

Qian Xu

Tian Xu

Tian Xu

Wang Xu

Zhou Xu-Jie

Yu Xuan

Zhao Xuan

Tian Xuebi

Deng Xuefeng

Guan Xuehai

Jiang Xuejuan

Wen Xuerong

Liu Xuhang

Bao Xuhui

Zhuang Xun

Zhao Xuna

Guo Xunyang

Lu Xuyang

Ozguler Y

Bai Ya Na

Kim Yae Jean

Herishanu Yair

Zhang Yajia

Chen Yalei

Dang Yalong

Takumi Yamada

Gong Yan

Huang Yan

Li Yan

Li Yan

Liu Yan

Ma Yan

Song Yan

Su Yan

Zhang Yan

Du Yang

Fan Yang

He Yang

Yang Yangfan

Shen Yanguang

Li Yaning

Ying Yanping

Ren Yanqin

Wang Yanqing

Zhang Yanqing

Liu Yanqiong

Wang Yanru

Lin Yansong

Yang Yanyan

Wang Yanzhou

Yu Yao

Hung Yao-Min

Tang Yaohui

Karatas Yasar

Cinar Yasin

Masaki Yasufumi

Tabara Yasuharu

Sugawara Yasuhiko

Sorimachi Yasunori

Fushimi Yasutaka

Feng Ye

Sun Ye

Yong Yean

Shoenfeld Yehuda

Chee Yen

Chen Yen-Shan

Wu Yen-Wen

Seo Yeon Seok

Ella Yeung

Cui Yi

Feng Yi

He Yi

Ji Yi

Li Yi

Liu Yi

Wang Yi

Wang Yi

Wang Yi

Zhang Yi

Zhou Yi

Lee Yi Che

Hsieh Yi-Chen

Chang Yi-Cheng

Wang Yi-Chian

Kuan Yi-Chun

Lu Yi-Fan

Huang Yi-Hsiang

Hsieh Yi-Hsien

Wang Yi-Jun

Lin Yi-Zhi

Zhou Yidi

Dang Yifang

Li Yifei

Chen Yihe

Chou Yiing-Jenq

Li Yijia

Liu Yijie

Zhu Yimin

Gu Ying

Liu Ying

Lu Ying

Luo Ying

Wang Ying

Wang Ying

Wen Ying

Lee Ying-Hsiang

Sun Yinghao

Tang Yinghua

Wu Yingli

Shi Yingtang

Li Yingxin

Yang Yinmo

Shen Yinzhong

Zhan Yiqiang

Xu Yixiang

Zhan Yiyang

Jiang Yizhou

Okubo Yoichiro

Nagashima Yoji

Kwong Yok Lam

Ozawa Yoko

Cai Yong

Liu Yong

Qiu Yong

Wang Yong

Zhao Yong

Lee Yong Chul

Kwon Yong Hwan

Kim Yong Kyun

Lee Yong-Ho

Yang Yong-Qing

Zhu Yong-Tong

Zhang Yongchun

Zhu Yonghong

Sun Yonghu

Dang Yonghui

Zhu Yongjin

Huo Yongliang

Fu Yonglun

Yang Yongping

Song Yoo Sung

Choi Yoonyoung

Chaiter Yoram

Yano Yoshihiko

Hayakawa Yoshihiro

Haga Yoshio

Naya Yoshio

Iwakura Yoshitsugu

Tanaka Yoshiya

Rosman Yossi

Luo You

Wang Youbin

Zhang Youjie

Chen Youjun

Chon Young Eun

Kim Young Hoon

Jo Young Suk

Kim Young-Hak

Kim Young-Kug

Oh Young-Min

An Young-Sil

Bae Younghyeon

Xiao Youping

Rezaei Yousef

Hu Yu

Liu Yu

Qian Yu

Fan Yu-Chen

Lee Yu-Chien

Li Yu-Chuan (Jack)

Huang Yu-Chuen

Lin Yu-Pang

Chang Yu-Ting

Chen Yu-Wei

Tien Yu-Wen

Huang Yu-Yao

Lin Yuan

Tian Yuan

Lee Yuan-Chieh

Lu Yuan-Qiang

Lu Yuan-Qiang

Wang Yuanjun

Wang Yuanyuan

Wang Yucai

Xie Yuchao

Qi Yuchen

Qiu Yudong

Fu Yue

Hu Yue

Huang Yue

Zhang Yue

Dou Yuetan

Sue Yuh-Mou

Li Yuhuang

Ishikawa Yuichi

Oike Yuichi

Huang Yujian

Tang Yujie

Ling Yun

Qiu Yun

Cheung Yun-Chung

Gai Yunchao

Chen Yung-Tai

Zhang Yunlong

Tang Yunneng

Lu Yunqi

Hu Yunwen

Mu Yunxiang

Yu Yunyong

Zhou Yunyun

Li Yuping

Zhang Yuping

Sun Yuqing

Feito Yuri

Wang Yushan

Jiang Yusheng

Harahsheh Yusra

Aydemir Yusuf

Fujiwara Yutaka

Liu Yuxuan

Miao Yuxuan

Calmus Yvon

Lucero Yvonne

Şahin Zafer

Bicakci Zafer

Imam Zaid

Long Zaiyang

Babic Zarko

Bogucki Zdzislaw

Zuo Zehua

Yueqin Zeng

Zhaochong Zeng

Walther Zenta

Dadaci Zeynep

Zhu Zezhang

Feng Zhanhui

Wang Zhanxiang

Dong Zhao

Sun Zhao

Meng Zhaowei

Li Zhaoyu

Qu Zhe

Wang Zhe

Yu Zhe

Zheng Zhe

Zhu Zhe

Meng Zhefeng

Cai Zhen

Ning Zhen

Qi Zhen

Guo Zhen-Ni

Ao Zheng

Tang Zheng

Deng Zhengrong

Qin Zhengtao

Qin Zhengtao

Chen Zhenguang

Fan Zhenqian

Li Zhentian

Wang Zhentian

Lu Zhenwei

Wen Zhi

Qiu Zhi-Bing

Cai Zhi-Gang

Zhao Zhi-Jing

Dai Zhi-Jun

Li Zhi-Jun

Xu Zhi-Xiang

Lv Zhibao

Ji Zhigang

Zhao Zhigang

He Zhiheng

Tan Zhijing

Cao Zhijuan

Gu Zhimin

Meng Zhipeng

Liang Zhipin

Sun Zhiqi

Cai Zhiyou

Zhang Zhongheng

Sun Zhonghua

Han Zhongji

Chen Zhonglei

Gao Zhongli

Xie Zhongqiu

Sun Zhongren

Yu Zhongxun

Li Zhuyun

Zhang Zhuzhu

Sabahat Zia

Zhao Zibo

Wu Ziheng

Chen Zijiang

Chen Zirong

Liu Ziying

Shaikh Zulfin

Wu Zunyou

Lu Zuxun

